# Hominids Lose Control

**DOI:** 10.1371/journal.pbio.0030073

**Published:** 2005-01-25

What makes us human? From a philosophical perspective, the answer may lie in part in our apparently unique need—and self-awareness—to ask the question in the first place. From a biological perspective, the answer lies in part in the sequence of our DNA. While fossil evidence has provided a rough draft of the story of human evolution, much more remains to be learned about the path our genes followed, a path that diverged millions of years ago from our closest living hominid relatives, the chimp and bonobo. Charting differences between human genomes and those of our evolutionary relatives—both near and distant—has become a powerful tool for filling in the gaps in the human fossil record.

Comparing the human genome to the genomes of other great apes can provide a window into the molecular changes that may ultimately spell the difference between human and nonhuman primates. That task was recently aided by the release of the draft sequence of the chimpanzee genome. Comparing the protein-coding sequences of human and chimp has identified molecular dissimilarities between us, which is to be expected. Though many differences between species can be explained at the molecular level by differences in protein structure, where and when a given protein is produced can be just as or even more important. Differences in protein expression arise from sequences in non-coding DNA that influence the timing and regulation of protein production and action.

In a new study, Peter Keightley and colleagues conduct parallel comparative genomics studies—comparing regulatory regions in the chimp and human genome with those of mouse and rat—and make a startling discovery. The hominid lineages show a surprising lack of selective constraint—deleterious mutations have apparently accumulated—compared to the rodents, racking up an estimated additional 140,000 harmful mutations fixed, or retained, in the human and chimp lineages since they diverged. Such mutations have been selectively eliminated in mouse and rat.

The authors focused on DNA sequences making up the bulk of gene-regulating elements—regions immediately preceding or following protein-coding sequences, as well as the first intron of each gene (an intron is a non-coding DNA sequence squeezed between two adjacent coding fragments). The degree of conservation in these areas was weighed against the conservation in other nearby non-coding sequences, which were assumed to be free of selective constraints. Keightley and colleagues found marked conservation in the regulatory regions between mice and rats, but nearly none between humans and chimps. This result suggests that the gene-regulating elements of hominids are subject to nearly unfettered mutation accumulation, likely due to an absence of natural selection forces strong enough to stabilize the ancestral sequences common to both human and chimpanzee.

How can one explain these puzzling results? Keightley and colleagues propose that selection is ineffective against mildly unfavorable mutations in the gene-regulating regions because of the small effective population size in the evolutionary history of hominids.

What do these results suggest for the future of human evolution? It's unlikely that the regulatory gatekeepers of our genome will allow mutations to spin out of control. Even if the number of unwanted mutations were to increase, stronger natural selection against them is likely to develop in parallel, Keightley and colleagues explain, protecting our fitness from a downward spiral. The authors' results support the notion that population size exerts a powerful influence on evolutionary changes at the molecular level and that many changes in gene control regions are under weak selection. With each new sequenced genome added to the comparative genomics lexicon, scientists are becoming increasingly conversant in the grammar and syntax of gene sequences—and filling in more and more gaps in the human story, letter by letter.

